# Generalizability of polygenic prediction models: how is the R^2^ defined on test data?

**DOI:** 10.1186/s12920-024-01905-8

**Published:** 2024-05-16

**Authors:** Christian Staerk, Hannah Klinkhammer, Tobias Wistuba, Carlo Maj, Andreas Mayr

**Affiliations:** 1 Department of Medical Biometry, Informatics and Epidemiology, Medical Faculty, University of Bonn, Bonn, Germany; 2 Institute of Statistics, RWTH Aachen University, Aachen, Germany; 3 Institute for Genomic Statistics and Bioinformatics, Medical Faculty, University of Bonn, Bonn, Germany; 4 Center for Human Genetics, University of Marburg, Marburg, Germany

**Keywords:** Polygenic risk scores, Prediction, Coefficient of determination, R squared, Calibration

## Abstract

**Background:**

Polygenic risk scores (PRS) quantify an individual’s genetic predisposition for different traits and are expected to play an increasingly important role in personalized medicine. A crucial challenge in clinical practice is the generalizability and transferability of PRS models to populations with different ancestries. When assessing the generalizability of PRS models for continuous traits, the $R^2$ is a commonly used measure to evaluate prediction accuracy. While the $R^2$ is a well-defined goodness-of-fit measure for statistical linear models, there exist different definitions for its application on test data, which complicates interpretation and comparison of results.

**Methods:**

Based on large-scale genotype data from the UK Biobank, we compare three definitions of the $R^2$ on test data for evaluating the generalizability of PRS models to different populations. Polygenic models for several phenotypes, including height, BMI and lipoprotein A, are derived based on training data with European ancestry using state-of-the-art regression methods and are evaluated on various test populations with different ancestries.

**Results:**

Our analysis shows that the choice of the $R^2$ definition can lead to considerably different results on test data, making the comparison of $R^2$ values from the literature problematic. While the definition as the squared correlation between predicted and observed phenotypes solely addresses the discriminative performance and always yields values between 0 and 1, definitions of the $R^2$ based on the mean squared prediction error (MSPE) with reference to intercept-only models assess both discrimination and calibration. These MSPE-based definitions can yield negative values indicating miscalibrated predictions for out-of-target populations. We argue that the choice of the most appropriate definition depends on the aim of PRS analysis — whether it primarily serves for risk stratification or also for individual phenotype prediction. Moreover, both correlation-based and MSPE-based definitions of $R^2$ can provide valuable complementary information.

**Conclusions:**

Awareness of the different definitions of the $R^2$ on test data is necessary to facilitate the reporting and interpretation of results on PRS generalizability. It is recommended to explicitly state which definition was used when reporting $R^2$ values on test data. Further research is warranted to develop and evaluate well-calibrated polygenic models for diverse populations.

**Supplementary Information:**

The online version contains supplementary material available at 10.1186/s12920-024-01905-8.

## Background

Genetics plays an increasingly important role in predicting disease risk and treatment responses. The development of polygenic risk scores (PRS) and general genetic prediction models for the individual liability to a particular clinical trait or phenotype comes with the promise to improve targeted early prevention and individual treatment [1–3]. However, an important limiting factor in current clinical practice is the issue of transferability of a developed PRS model to out-of-target populations [4–7]. This especially concerns the application of PRS in populations that have been underrepresented in the cohorts used to estimate and train the models, such as those with genetic ancestry different from the majority of participants in current cohorts. As many current PRS models are predominantly trained and optimized on individuals with European ancestry, this disparity also raises important ethical issues [8].

Amid these challenges, PRS work by quantifying the genetic predisposition for a particular trait and are typically computed using weighted sums of risk alleles, where the weights are based on effect sizes that represent associations between variants and the target phenotype [9]. The effect sizes of individual variants (single nucleotide polymorphisms, SNPs) are often small for common traits characterized by a polygenic architecture. As a consequence of differences in allele frequencies and linkage disequilibrium patterns (i.e., variant correlations) across populations, absolute PRS values may be influenced more by population structure than by genuine genetic risk for the phenotype [10]. Such issues should be taken into account when evaluating the transferability of PRS models to out-of-target populations. This is particularly important when the ultimate aim is the development of individual prediction models for personalized medicine, integrating the PRS with further demographic and environmental variables.

The evaluation of polygenic prediction models on test data plays an essential role to examine the generalizability and transferability to different populations. However, even though it may sound straightforward, there is no general consensus on the choice of the evaluation metric [11–14]. The methodological distinction between discrimination and calibration is a well-known issue in the context of prediction models for binary outcomes [15, 16]. While the AUC is the most widely used measure for evaluating the prediction accuracy of polygenic models for binary outcomes, it solely reflects discriminative accuracy. To assess also calibration, additional measures such as the Brier score and graphical tools such as calibration plots are recommended [17, 18]. When we focus on the prediction of a continuous trait, it seems obvious that the predicted values should be as close as possible to the true (observed) values. But how should this closeness be measured, particularly when there is a test sample of $n_{\text {test}}$ observations? Researchers working in statistics might answer with the mean squared prediction error (MSPE) or its root (RMSPE), which take both bias and variance of predictions into account. If there are outliers in the data, other researchers might want to focus on the more robust median absolute error (MAE), while researchers working with the actual predictions might argue that both MSPE and MAE do not provide results that are easily interpretable or comparable across different outcomes and might focus on percentage errors (e.g., the median absolute percentage error).

In the context of polygenic risk modelling (but not only there [14]), a popular measure of prediction accuracy for continuous traits is the $R^2$ [19–22]. The $R^2$ in statistics is typically a measure for the goodness-of-fit of a model on the training data and is also called the coefficient of determination. In the context of linear models, it is a well-defined measure on training data and can be interpreted as the proportion of variability of the outcome explained by the model. However, in case of using the $R^2$ for predictions on test data the situation is generally less clear and the $R^2$ measure is not unambiguously defined. In particular, one potential definition of the $R^2$ is simply based on the squared correlation between predicted and observed values, which is always bounded between 0 and 1. This definition makes the $R^2$ relatively easy to interpret and to compare between different phenotypes (similar to the AUC for binary outcomes). Two alternative definitions of the $R^2$ are closer to the original definition on training data (cf., [23]), comparing the MSPE of the prediction model in relation to an intercept model (one based on training data, one based on test data). In contrast to the squared correlation which only focuses on the discriminative performance, the MSPE-based definitions of the $R^2$ take both discrimination and calibration of the prediction model into account.

In this study, we investigate the impact of these three different $R^2$ definitions when analysing the accuracy of PRS models on test data and illustrate their differences in the context of the generalizability to different populations. Our UK Biobank data analysis shows that phenotype predictions of standard PRS models on out-of-target populations are often considerably biased, which can even lead to negative $R^2$ values based on the two MSPE-based definitions, strongly indicating miscalibrated predictions. On the other hand, the (squared) correlation with the phenotype may partially be preserved in some cases even when the PRS is systematically biased and poorly calibrated, as it only addresses the discriminative performance. As a consequence, resulting $R^2$ values from the different definitions lead to considerably different results — not only regarding their absolute values, but also when using the $R^2$ to rank different PRS models developed by competing methods. We highlight the strengths and limitations of the different $R^2$ definitions and discuss recommendations regarding their reporting.

## Methods

### Different $R^2$ definitions on test data

The coefficient of determination $R^2$ was originally introduced as a goodness-of-fit measure for linear models [24] and can be calculated in various but equivalent ways due to the variance decomposition [25]. When applied to training data in linear models, the $R^2$ can be interpreted as the proportion of variance that is explained by the model. However, the variance decomposition does not generally hold true on test data or for non-linear models, leading to different results depending on the choice of $R^2$ formula. While the different formulas for the $R^2$ on training can lead to various ways to define the $R^2$ on test data [25], here we focus on the three most common definitions.

The first definition is given by the squared correlation $r^2$ between the observed phenotypes $\varvec{y}=(y_1,\dots ,y_{n_\text {test}})$ and the predictions $\varvec{\hat{y}}=(\hat{y}_1,\dots ,\hat{y}_{n_\text {test}})$, i.e.,

1 $$\begin{aligned} r^2{} & {} = \text {cor}(\varvec{y},\varvec{\hat{y}})^2 \nonumber \\{} & {} =\frac{\left( \sum _{i=1}^{n_\text {test}}(y_i-\bar{y})(\hat{y}_i-\bar{\hat{y}})\right) ^2}{\sum _{i=1}^{n_\text {test}}\left( y_i-\bar{y}\right) ^2\sum _{i=1}^{n_\text {test}}\left( \hat{y}_i-\bar{\hat{y}}\right) ^2}, \end{aligned}$$

where ${n_\text {test}}$ is the number of individuals in the test set, and $\bar{y}$ and $\bar{\hat{y}}$ are the means of the observed and predicted phenotypes, respectively. Noteworthy, $r^2$ based on Eq. (1) is always nonnegative and bounded between 0 and 1. This is the same as fitting a simple linear model with intercept on the test data with the PRS model predictions as the only covariate and computing the classical coefficient of determination for this model. The $r^2$ value tends to be high in the presence of a strong linear correlation, regardless of whether the predictions are strongly biased and poorly calibrated. In fact, the association could even be negative. Therefore, when considering the squared correlation $r^2$, it is recommended to additionally examine scatterplots and the correlation coefficient *r* itself.

Two alternative definitions of the $R^2$ on test data, which are closer to the original model-comparison idea of $R^2$ (cf., [23, 26]), are based on relative comparisons of the out-of-sample residual sum of squares (or, equivalently, the MSPE) for the prediction model in relation to a reference model. In contrast to the squared correlation $r^2$, which only evaluates the discrimative performance of the model, the two MSPE-based definitions of the $R^2$ consider both discrimination and calibration of the prediction model. In case (A), the intercept model on the training data is used as a reference, leading to the definition

2 $$\begin{aligned} R^2_{1-\text {A}}= 1-\frac{\sum _{i=1}^{n_\text {test}}\left( y_i-\hat{y}_i\right) ^2}{\sum _{i=1}^{n_\text {test}}\left( y_i-\bar{y}_\text {train}\right) ^2} , \end{aligned}$$

where $\bar{y}_\text {train}$ is the mean of the phenotype in the training data (representing the intercept model).

In case (B), the intercept model on the test data is used as a reference, leading to the definition

3 $$\begin{aligned} R^2_{1-\text {B}}= 1-\frac{\sum _{i=1}^{n_\text {test}}\left( y_i-\hat{y}_i\right) ^2}{\sum _{i=1}^{n_\text {test}}\left( y_i-\bar{y}_\text {test}\right) ^2} , \end{aligned}$$

where $\bar{y}_\text {test}$ is the mean of the phenotype in the test data (representing the intercept model on test data).

Both MSPE-based definitions, $R^2_{1-\text {A}}$ and $R^2_{1-\text {B}}$, have an upper bound of 1 with equality in case of perfect predictions on test data, i.e., $R^2_{1-\text {A}}=R^2_{1-\text {B}}=1$ if and only if

$$\begin{aligned} \text {MSPE}=\frac{1}{n_\text {test}}\sum \limits _{i=1}^{n_\text {test}}\left( y_i-\hat{y}_i\right) ^2=0 . \end{aligned}$$

On the other hand, $R^2_{1-\text {A}}$ and $R^2_{1-\text {B}}$ are generally not lower bounded and can even be negative, particularly for prediction models that are miscalibrated on the test data (see Results section). If $R^2_{1-\text {A}}$ is negative, this implies that a simple intercept-only model on the training data yields a smaller MSPE on the test data than the PRS model. In a similar vein, if $R^2_{1-\text {B}}$ is negative, this means that a simple intercept model on the test data performs better than the PRS model.

Note that in situations where the size of the test data is small, considering the mean on the test data leads to increased variability of the $R^2_{1-\text {B}}$ measure, and the $R^2_{1-\text {A}}$ definition may generally be preferable based on theoretical reasons [23]. Yet, while $r^2$ and $R^2_{1-\text {B}}$ only depend on the predicted and observed phenotypes on the test data, $R^2_{1-\text {A}}$ also explicitly depends on the training data, implying that $R^2_{1-\text {A}}$ cannot be readily computed when the phenotype mean on the training data is not available.

### Data

All analyses were performed on data from the UK Biobank (UKBB) under application number 81202. The UKBB [27] is a large-scale prospective cohort study of approximately 500,000 participants from the United Kingdom, established in 2006. Participants were aged between 40 and 69 years at recruitment. Resources include individual-level genotype data and detailed information on a wide range of phenotypes for each participant, such as physical measurements as well as blood biomarkers. Baseline data are continuously updated through follow-up studies.

We extracted data for the phenotypes height (UKBB field 50), BMI (UKBB field 21001) and lipoprotein A (UKBB field 30790), as well as for the covariates sex (UKBB field 22001), age (UKBB field 21022) and the first ten principal components (PCs, UKBB field 22009) of the genotype matrix. As performed in other works, individual genetic ancestry was derived considering the closest reference population center (geometric medians) according to the Euclidean distance in the global PC space based on 1K genome data [28]. Incomplete data, related individuals as well as individuals who could not be assigned to European, African, East Asian and South Asian population clusters, were discarded, resulting in $n=333{,}587$ individuals comprising $n=319{,}456$ of European ancestry, $n=5{,}411$ of African ancestry, $n=6{,}796$ of South Asian ancestry and $n=1{,}924$ of East Asian ancestry. The European subcohort was further split into training ($n=159{,}554$), validation ($n=80{,}113$) and test set ($n=79{,}799$). Genotype data (UKBB category 100315) were filtered for a genotype rate of at least 90% and a minor allele frequency of at least 0.1%, resulting in $p=549{,}426$ SNPs.

### Computation of PRS

PRS models for each phenotype were derived based on the training and validation sets of the European subcohort via three competing estimation approaches: PRScs [29], snpboost [30] and BayesR [31]. While PRScs applies Bayesian shrinkage on univariate effect estimates from GWAS summary statistics, snpboost and BayesR estimate PRS directly based on individual-level genotype data, using statistical boosting and Bayesian hierarchical modelling, respectively.

To apply PRScs, first a GWAS was conducted in plink2 [32, 33] on the European training set. Afterwards, the European validation set as well as the LD reference based on UKBB data (provided at https://github.com/getian107/PRScs) were used to run PRScs and derive the final weights. For BayesR, which also applies Bayesian shrinkage on the effect estimates but works on individual-level data, the implementation in LDAK [34] was used. Per-predictor heritabilities were computed assuming the BLD-LDAK-model and incorporating the GWAS results based on the training set. Using those per-predictor heritabilities, BayesR was run on the European training set and the final model was chosen to optimize the performance on the European validation set. The statistical boosting algorithm snpboost was applied on individual-level data. Effect estimates by snpboost were derived on the European training set incorporating early stopping based on the predictive performance on the validation set to provide sparse models.

Finally, PRS were computed for all individuals using plink’s --score function based on the estimated weights from the three different methods. While snpboost directly provides prediction models for the phenotype of interest, PRScs and BayesR only yield scores that must be rescaled to predict the phenotype. For a fair comparison, for all three methods a multivariable regression was fitted on the combined European training and validation data including the PRS, sex, age and the first ten principal components as predictors. The resulting models were then applied on the test data set comprising individuals of European, African, East Asian and South Asian ancestry.

Additionally, we conducted two sensitivity analyses regarding different variant filtering and adjustment for population stratification. In a first sensitivity analysis, we used more stringent variant filters, i.e., a minor allele frequency filter of 1% and a genotype call rate of at least 99%. In a second sensitivity analysis, we included the first 10 genetic PCs directly in the GWAS and in the training of snpboost.

## Results

**Fig. 1:**
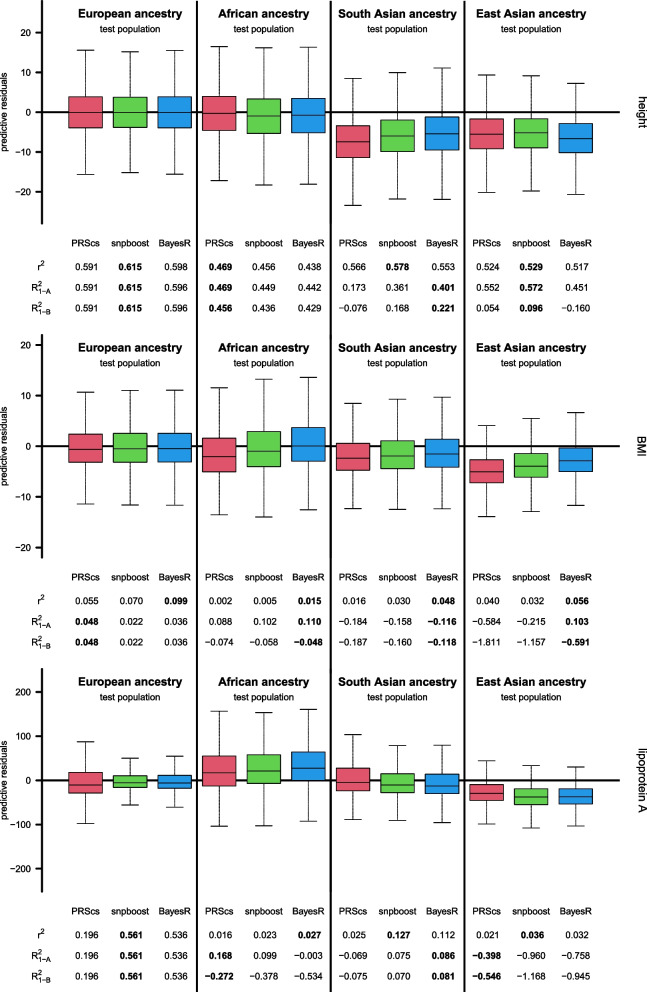
Boxplots of predictive model residuals for the prediction of height (top), BMI (middle) and lipoprotein A (bottom) on test populations with different ancestries, together with $R^2$ values based on the three different definitions in Eqs. (1), (2) and (3). Different polygenic prediction models (including sex, age and first 10 genetic PCs) were derived on training data with European ancestry using PRScs, snpboost and BayesR, respectively. Outliers are not shown in the boxplots

Figure 1 provides results for the three different definitions of the $R^2$ measure on European, African, South Asian and East Asian ancestry test populations for the prediction of height, BMI and lipoprotein A on UK Biobank data. Results are based on PRS models derived by PRScs [29], snpboost [30] and BayesR [31] on the European training population (including the covariates sex, age and the first 10 genetic PCs). Sensitivity analyses regarding different variant filtering and adjustment for population stratification lead to overall comparable results and main conclusions (see Supplementary Figure 1 and Supplementary Figure 2).

The three considered phenotypes, height, BMI and lipoprotein A, exhibit a significant genetic component, but present distinct underlying genetic architectures. As such, they are well-suited for evaluating diverse scenarios, specifically regarding different $R^2$ definitions and competing PRS models with varying degrees of sparsity. In particular, height and BMI are highly polygenic traits [35]; however, while the environmental influence remains minimal for height [36], a substantial portion of the genetic signal in BMI can be attributed to gene-environment interactions [37]. As we do not consider environmental variables in this work, the predictive performance of the PRS models are particularly limited for BMI, with relatively low $R^2$ values observed for the different models and definitions even on the European test population (see Fig. 1). Conversely, lipoprotein A blood levels are predominantly influenced by an oligogenic component, with the majority of the signal originating from variants located within the LPA gene locus [38]. The predictive performance of the competing methods shown in Fig. 1 clearly reflect the sparse genetic architecture of lipoprotein A, as the sparse PRS models derived by snpboost and BayesR yield considerably improved performance compared with the non-sparse model derived by PRScs [29, 30].

On the European test population, the predictive residuals are approximately centered around zero for all PRS methods (Fig. 1), with symmetric residual distributions for height, while for BMI and lipoprotein A the median residuals are negative, reflecting the right-skewed distributions of these phenotypes. On the European test population, the three different definitions of $R^2$ lead to quite similar values, especially for height and lipoprotein A, where the models derived by snpboost consistently show the best performance across all $R^2$ definitions. However, already on the European test population, the three definitions of $R^2$ are not generally equivalent on test data, as can be seen for the case of BMI, where PRScs performs best according to the MSPE-based definitions $R^2_{1-\text {A}}$ and $R^2_{1-\text {B}}$ (PRScs: $R^2_{1-\text {A}}=0.048$, BayesR: $R^2_{1-\text {A}}=0.036$, snpboost: $R^2_{1-\text {A}}=0.022$), while BayesR performs best according to the squared correlation $r^2$ between predicted and observed BMI (BayesR: $r^2=0.099$, snpboost: $r^2=0.070$, PRScs: $r^2=0.055$). Note that the rankings of different prediction models developed on the same training data are always the same based on the maximization of $R^2_{1-\text {A}}$ or $R^2_{1-\text {B}}$ as well as based on the minimization of the MSPE, as both $R^2_{1-\text {A}}$ and $R^2_{1-\text {B}}$ can just be viewed as different ways of “rescaling” the MSPE.

Figure 1 further reveals that, on test populations with African, South Asian and East Asian ancestry, the distributions of predictive residuals tend to be shifted and not centered around zero, indicating miscalibration of the PRS models. In such situations, the three different definitions of $R^2$ on test data can lead to considerably different results. For example, for height in the South Asian ancestry population, the distributions are shifted towards negative residuals, reflecting the overestimation on the South Asian population based on the PRS models that were developed on the European training population. Considering the $R^2$ definition based on the squared correlation between predicted and observed heights still yields relatively large values for the different PRS models on the South Asian population (between $r^2=0.578$ for snpboost and $r^2=0.553$ for BayesR), while the MSPE-based definitions $R^2_{1-\text {A}}$ and $R^2_{1-\text {B}}$ yield considerably lower and potentially even negative values (between $R^2_{1-\text {A}}=0.401$, $R^2_{1-\text {B}}=0.221$ for BayesR and $R^2_{1-\text {A}}=0.173$, $R^2_{1-\text {B}}=-0.076$ for PRScs). Therefore, for the prediction of height on the South Asian test population, snpboost performs best according to the definition based on the squared correlation $r^2$, while BayesR performs best according to the MSPE-based definitions $R^2_{1-\text {A}}$ and $R^2_{1-\text {B}}$.

**Fig. 2:**
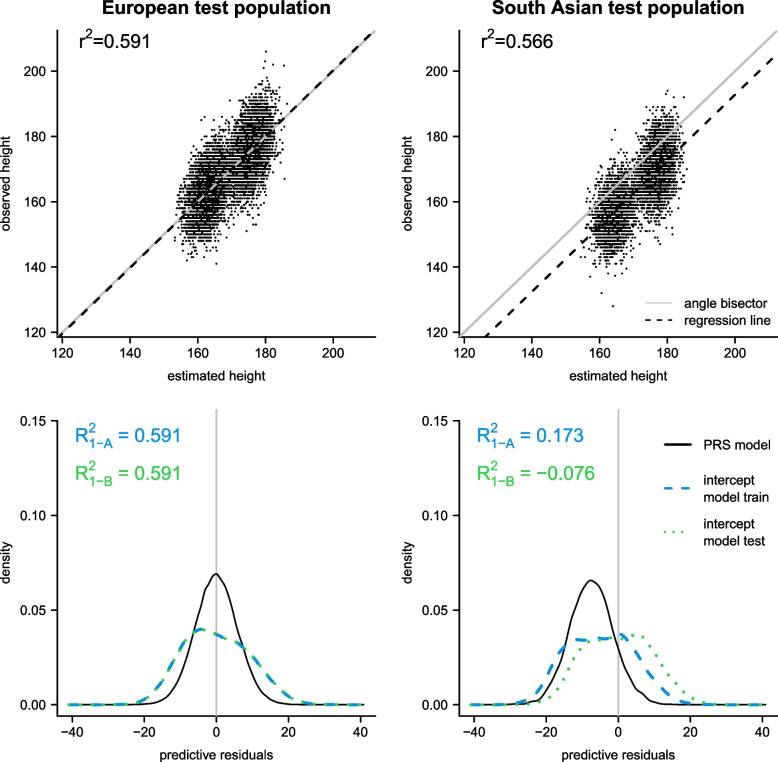
Illustration of the three different $R^2$ definitions on test populations with European ancestry (left) and South Asian ancestry (right) for the prediction of height, based on PRScs model derived on training data with European ancestry (including sex, age and first 10 genetic PCs). The scatterplots (top) show the association between predicted and observed heights, illustrating the $R^2$ definition based on the squared correlation ($r^2$). The kernel density plots (bottom) show the distributions of predictive residuals, illustrating the MSPE-based definitions of the $R^2$ with reference to intercept models on training data ($R^2_{1-\text {A}})$ and test data ($R^2_{1-\text {B}})$, respectively

Figure 2 illustrates the underlying reasons for the conflicting results of the three different definitions of the $R^2$ measure for the prediction of height, focusing on the PRS model derived by PRScs. The two scatterplots (first row in Fig. 1) indicate relatively strong correlations between the predicted heights from the PRS model and the observed (true) heights on both the European and South Asian test populations. In line with this, the prediction performance according to the $R^2$ definition based on the squared correlation between predicted and observed heights appears to be only slightly worse on the South Asian ancestry population ($r^2=0.566$) compared to the European population ($r^2=0.591$). However, while for the European test population the regression line between predicted and observed heights lies on the angle bisector, a clear parallel shift of the regression line is apparent for the test population of South Asian ancestry, indicating a systematic overestimation of height by the PRS model on this population.

The miscalibration of the predictions on the South Asian test population becomes even clearer when inspecting kernel density plots for the distributions of predictive residuals (observed height – predicted height), see second row in Fig. 2. While for the European test population the predictive residuals of the PRS model are centered around zero, the distribution of predictive residuals is shifted towards negative residuals (i.e., smaller observed heights than predicted by the PRS model) for the South Asian test population. In contrast to the definition based on the squared correlation $r^2$, the two MSPE-based definitions $R^2_{1-\text {A}}$ and $R^2_{1-\text {B}}$ are sensitive to the miscalibration of the PRS model on the South Asian population. In particular, while on the European test population the two MSPE-based definitions of $R^2$ yield similar values to the squared correlation, both MSPE-based definitions yield considerably lower values on the population with South Asian ancestry ($R^2_{1-\text {A}} = 0.173, R^2_{1-\text {B}} = -0.076$ and $r^2=0.566$).

Recall that the definition $R^2_{1-\text {A}}$ relates the MSPE of the PRS model to the MSPE of the intercept model derived on the training data (see Eq. 2), while the definition $R^2_{1-\text {B}}$ relates the MSPE of the PRS model to the variance of observed heights on the test data (see Eq. 3). Therefore, as the distribution of the PRS model residuals on the South Asian population is slightly “closer” (on average in terms of squared distance) to zero compared to the distribution of the predictive residuals from the intercept model derived on the training data (shown in blue), the value of $R^2_{1-\text {A}} = 0.173$ is still positive. On the other hand, when compared to the intercept model on the South Asian test population (i.e., corresponding to the variance of observed heights, shown in green), the PRS model predictive residuals show a larger variability around zero, so that the value of $R^2_{1-\text {B}} = -0.076$ is negative. This example illustrates that the MSPE-based definitions can be positive or negative on test data depending on the performance of the prediction model relative to the intercept model on training data ($R^2_{1-\text {A}}$) or test data ($R^2_{1-\text {B}}$), whereas the definition based on the squared correlation ($r^2$) is guaranteed to always yield nonnegative values. Additional scatterplots and distributions of predictive residuals (as in Fig. 2) for the test populations of African and East Asian ancestries are shown in the Supplementary Material (see Supplementary Figures 3 and 4, respectively). For the East Asian population, it is apparent that a larger difference between phenotype means in the training and test data can lead to considerably different $R^2_{1-A}$ and $R^2_{1-B}$ values, whereas the distributions of the predictive residuals derived from the intercept models almost coincide for the population of African ancestry leading to similar $R^2_{1-A}$ and $R^2_{1-B}$ values.

Figure 1 shows substantial differences in the three definitions of $R^2$ on test data not only for height but also for the other phenotypes on out-of-target populations. While for height the squared correlations $r^2$ between predicted and observed phenotypes still remain relatively large (cf., Fig. 2), for lipoprotein A the prediction accuracy, even in terms of $r^2$, is considerably reduced for populations with non-European ancestry. This indicates that the loss of prediction accuracy of the PRS models for lipoprotein A on out-of-target populations is not only a matter of miscalibration.

## Discussion

Although the $R^2$ is a widely-used and apparently simple measure, it is not clearly defined for the evaluation of prediction models on test data. In our analysis on the UK Biobank, we have illustrated that there are at least three alternative definitions of the $R^2$ on test data that lead to considerably different results when used for the evaluation of the generalizability of PRS models to out-of-target populations (Fig. 1). In particular, we have shown that these different definitions can also lead to conflicting rankings of competing prediction methods. Therefore, to ensure reproducibility and fair comparisons between methods, we strongly recommend explicitly stating which definition was used when reporting $R^2$ values.

Our study does not aim to determine which definition of the $R^2$ should be considered as the *correct* one on test data, since all of them have their advantages and challenges. Yet, we still want to provide some general advice on which might be the most suitable definition depending on the data situation and modelling aims. If the PRS is aimed to be primarily used as a stratification tool, similar to other biomarkers, then the definition based on the squared correlation $r^2$ between predicted and observed outcomes might be preferred, as it implicitly assumes a re-calibration when used on another population. On the other hand, if the PRS model should be used as a direct prediction model for continuous phenotypes of individuals, then the squared correlation $r^2$ cannot capture miscalibration and the MSPE-based definitions $R^2_{1-A}$ and $R^2_{1-B}$ might be more suitable. Unlike the squared correlation $r^2$, the MSPE-based definitions can also take on negative values. This property should generally not be viewed as a deficiency of the MSPE-based definitions, since negative values of $R^2_{1-A}$ or $R^2_{1-B}$ are important indicators of poor calibration, showing that the PRS model on the test population is inferior to a basic intercept model.

In this context, one can argue that the $R^2_{1-A}$ definition, which relies on the arithmetic mean on the training data (or the intercept model on the training population), is generally preferable compared to the $R^2_{1-B}$ definition relying on the mean on the test data, particularly in cases where the size of the test data is small leading to increased variability of the $R^2_{1-B}$ measure [23]. On the other hand, the comparison of different PRS models that might have been derived based on different training cohorts is not feasible with the $R^2_{1-A}$ definition in situations where the means on the different training data are not available. Therefore, in such cases, the $R^2_{1-B}$ definition can be considered a pragmatic alternative choice. Furthermore, note that the $R^2_{1-B}$ definition can also be regarded as “more conservative”: to obtain a positive $R^2_{1-B}$ value, the PRS model needs to provide better predictions than the mean on the population it is tested on.

In practice, it can be valuable to consider both the correlation-based and the MSPE-based definitions as they provide complementary information. In particular, when the squared correlation $r^2$ is high, but the MSPE-based measures $R^2_{1-A}$ or $R^2_{1-B}$ are low, this indicates that a well-calibrated new prediction model on the targeted test population may be achieved after using an appropriate affine transformation of the original model predictions. On the other hand, when both the correlation-based and MSPE-based measures are low for a PRS model on a particular population, the poor discrimination already indicates that the PRS model may generally not be suitable for the targeted population. As the squared correlation $r^2$ between predicted and observed phenotypes only assesses the discriminatory accuracy of prediction models, to investigate also the calibration of PRS models for continuous phenotypes, we recommend to use additional measures such as the MSPE or the MSPE-based definitions of the $R^2$, as well as graphical tools such as scatterplots as in Fig. 2 (cf., [13]). This is especially important when considering out-of-target populations.

In this study, we evaluated PRS accuracy using $R^2$ population-level metrics on test data based on individuals with different genetic ancestries, for which we employed genetic principal component clustering based on the reference 1K Genome superpopulations [39]. Considering population clusters is an important limitation, as human genetic diversity extends across a continuum, and even populations deemed “homogeneous” exhibit accuracy variations along subcontinental ancestries, influenced by the genetic distance from training data [40]. Our analysis relying on population clusters hence simplifies the underlying genetic heterogeneity. Nevertheless, this approach is suitable for comparing $R^2$ performance among categorical groups, identifying reporting biases that would actually emerge within a continuous spectrum. Our results further emphasize the importance of comprehensive global human genome studies to effectively capture genetic heterogeneity, with the goal of offering more universally applicable genetic risk scores. Another limitation of our study is that we focused on state-of-the-art methods to estimate PRS models on a training population with European ancestry; these methods are not specifically designed to provide robust generalizability to out-of-target populations. While more refined methods might be able to provide improved transferability of the resulting PRS models (see [6] for a comprehensive review of recent methods), the issues we discussed regarding the different definitions of the $R^2$ on test data are still highly pertinent for the evaluation of such models.

The transferability of prediction models remains one of the most important challenges in the practical application of PRS and its implementation in clinical routines and decision rules. For PRS or other genetic scores to play a pivotal role in future public health and disease prevention programs, it is imperative that individuals with non-European ancestry and multi-ethnic backgrounds benefit equally from these tools, just as individuals with European ancestry. Further research is warranted on broader and more diverse cohorts as well as new methods that help to close the substantial gap in accuracy that can be observed in practice. For this purpose, it is essential to have clear and transparent ways to evaluate new methods for the development of PRS also on other cohorts.

## Conclusions

In this article, we have investigated three different definitions of the $R^2$ measure on test data and illustrated how these behave when evaluating the accuracy of European-derived PRS models that were applied to predict continuous phenotypes on individuals with different ancestry. The choice of the $R^2$ definition on test data might drastically influence the results, particularly when evaluating the prediction performance on out-of-target populations. If the PRS model is primarily aimed for risk stratification, then defining the $R^2$ as the squared correlation between predicted and observed phenotypes provides the clearest interpretation. If the PRS model is also targeted for individual predictions, model calibration is crucial, which can be assessed via graphical tools and additional measures, including the MSPE-based definitions of the $R^2$. To ensure reproducibility and fair comparisons with results from the literature, it is essential to state which definition was used when reporting $R^2$ measures on test data.

### Supplementary Information

Supplementary Material 1.

## Data Availability

The data analyzed in this study is subject to the following licenses/restrictions: This research has been conducted using the UK Biobank resource under application number 81202 (http://www.ukbiobank.ac.uk). Requests to access these datasets should be directed to UK Biobank, http://www.ukbiobank.ac.uk.

## Abbreviations

AUC: Area under the curve
BMI: Body mass index
GWAS: Genome-wide association study
MAE: Mean absolute error
MSPE: Mean squared prediction error
PC: Principal component
PRS: Polygenic risk score
SNP: Single nucleotide polymorphism
UKBB: UK Biobank
