# Supplementary material for: Generalizability of polygenic prediction models: how is the R2 defined on test data?
Source: BMC Med Genomics. 2024 May 16;17:132. doi: 10.1186/s12920-024-01905-8 (PMC11100126; doi:10.1186/s12920-024-01905-8)
Supplement: Supplementary file 1 — Supplementary Material 1. [file 12920_2024_1905_MOESM1_ESM.pdf]

Supplementary Material

Generalizability of polygenic prediction models: How is the R^2 defined on test data?

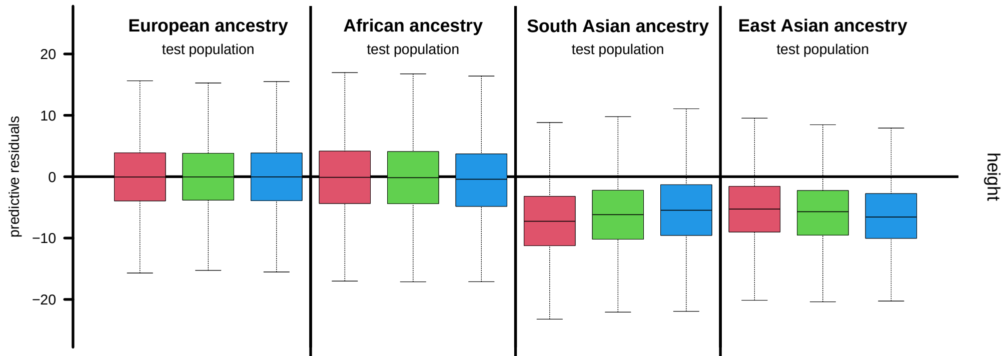

	PRScs	snpboost	BayesR	PRScs	snpboost	BayesR	PRScs	snpboost	BayesR	PRScs	snpboost	BayesR
r^2	0.591	**0.613**	0.601	**0.470**	0.456	0.441	0.570	**0.574**	0.556	0.523	**0.526**	0.519
R^2_{1-A}	0.591	**0.613**	0.599	**0.471**	0.461	0.449	0.205	0.332	**0.394**	**0.564**	0.524	0.457
R^2_{1-B}	0.591	**0.613**	0.599	**0.459**	0.448	0.436	−0.035	0.131	**0.212**	**0.078**	−0.006	−0.148

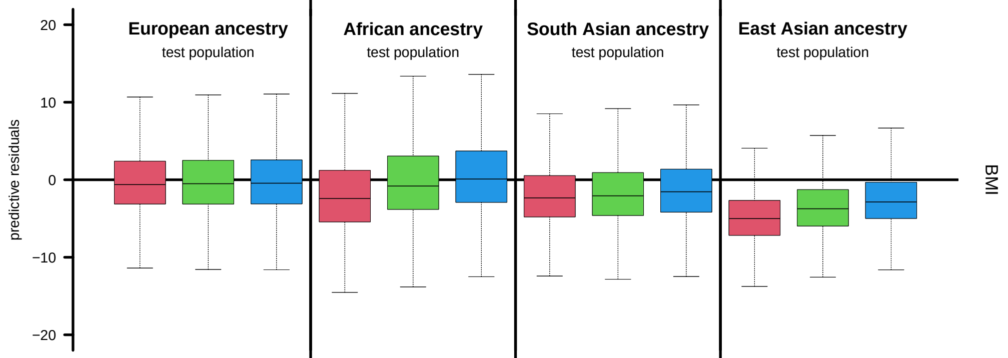

	PRScs	snpboost	BayesR	PRScs	snpboost	BayesR	PRScs	snpboost	BayesR	PRScs	snpboost	BayesR
r^2	0.055	0.072	**0.099**	0.002	0.005	**0.015**	0.015	0.030	**0.048**	0.044	0.033	**0.056**
R^2_{1-A}	**0.048**	0.025	0.036	0.056	0.105	**0.109**	−0.194	−0.193	**−0.119**	−0.561	−0.161	**0.106**
R^2_{1-B}	**0.048**	0.025	0.036	−0.112	−0.054	**−0.050**	−0.196	−0.196	**−0.121**	−1.770	−1.061	**−0.586**

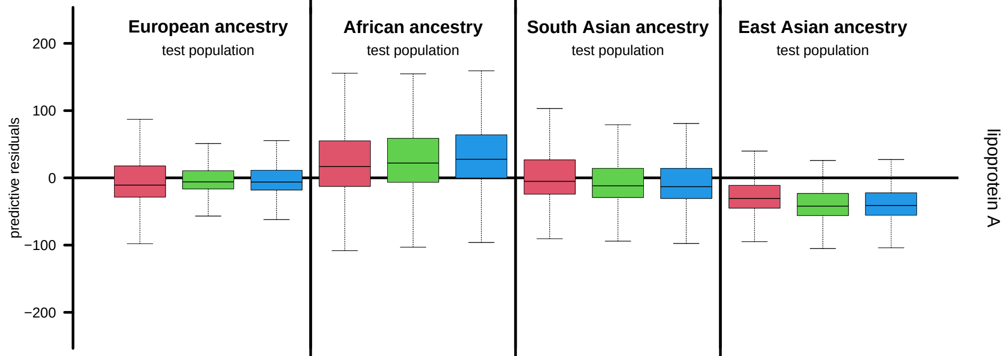

	PRScs	snpboost	BayesR	PRScs	snpboost	BayesR	PRScs	snpboost	BayesR	PRScs	snpboost	BayesR
r^2	0.195	**0.552**	0.530	0.016	0.018	**0.027**	0.026	**0.124**	0.107	0.031	**0.059**	0.041
R^2_{1-A}	0.195	**0.552**	0.530	**0.170**	0.074	−0.005	−0.060	0.068	**0.076**	**−0.318**	−0.801	−0.714
R^2_{1-B}	0.195	**0.552**	0.529	**−0.269**	−0.417	−0.537	−0.066	0.063	**0.071**	**−0.459**	−0.992	−0.897

Supplementary Figure 1: Boxplots of model residuals for the prediction of height (top), BMI (middle) and lipoprotein A (bottom) on test populations with different ancestries, together with R^2 values based on the three different definitions in equations (1), (2) and (3) of the paper. Outliers are not shown in the boxplots. In contrast to Figure 1, a genotype rate filter of 99% and a minor allele frequency filter for 1% were used.

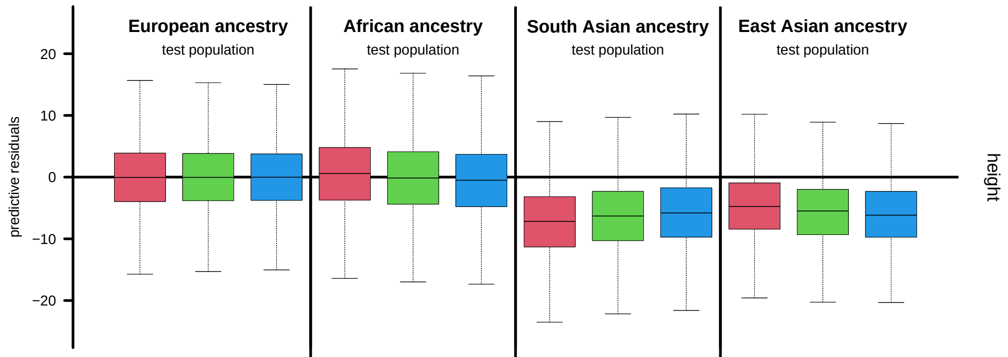

	European PRScs	European snpboost	European BayesR	African PRScs	African snpboost	African BayesR	South Asian PRScs	South Asian snpboost	South Asian BayesR	East Asian PRScs	East Asian snpboost	East Asian BayesR
r^2	0.589	0.613	**0.623**	**0.471**	0.457	0.448	0.558	0.573	**0.575**	0.522	0.523	**0.535**
R^2_{1-A}	0.589	0.612	**0.622**	**0.468**	0.462	0.452	0.199	0.318	**0.377**	**0.605**	0.543	0.501
R^2_{1-B}	0.589	0.612	**0.622**	**0.455**	0.449	0.439	−0.043	0.113	**0.189**	**0.166**	0.033	−0.054

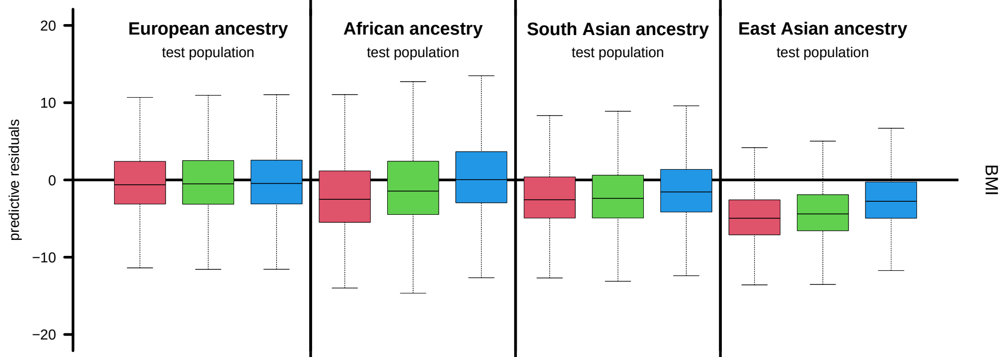

	European PRScs	European snpboost	European BayesR	African PRScs	African snpboost	African BayesR	South Asian PRScs	South Asian snpboost	South Asian BayesR	East Asian PRScs	East Asian snpboost	East Asian BayesR
r^2	0.055	0.071	**0.101**	0.003	0.006	**0.015**	0.016	0.029	**0.049**	0.039	0.034	**0.057**
R^2_{1-A}	**0.048**	0.025	0.042	0.051	0.095	**0.112**	−0.226	−0.251	**−0.114**	−0.531	−0.378	**0.120**
R^2_{1-B}	**0.048**	0.025	0.042	−0.118	−0.066	**−0.047**	−0.228	−0.253	**−0.116**	−1.718	−1.446	**−0.562**

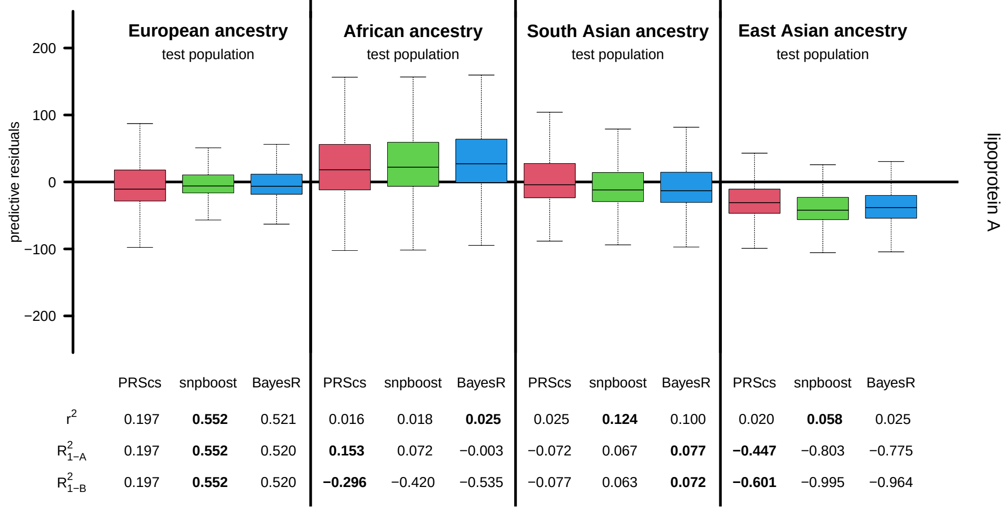

	European PRScs	European snpboost	European BayesR	African PRScs	African snpboost	African BayesR	South Asian PRScs	South Asian snpboost	South Asian BayesR	East Asian PRScs	East Asian snpboost	East Asian BayesR
r^2	0.197	**0.552**	0.521	0.016	0.018	**0.025**	0.025	**0.124**	0.100	0.020	**0.058**	0.025
R^2_{1-A}	0.197	**0.552**	0.520	**0.153**	0.072	−0.003	−0.072	0.067	**0.077**	**−0.447**	−0.803	−0.775
R^2_{1-B}	0.197	**0.552**	0.520	**−0.296**	−0.420	−0.535	−0.077	0.063	**0.072**	**−0.601**	−0.995	−0.964

Supplementary Figure 2: Boxplots of model residuals for the prediction of height (top), BMI (middle) and lipoprotein A (bottom) on test populations with different ancestries, together with R^2 values based on the three different definitions in equations (1), (2) and (3) of the paper. In contrast to Figure 1, the genetic PCs were directly used in the GWAS underlying PRScs and BayesR, as well as in the training of snpboost.

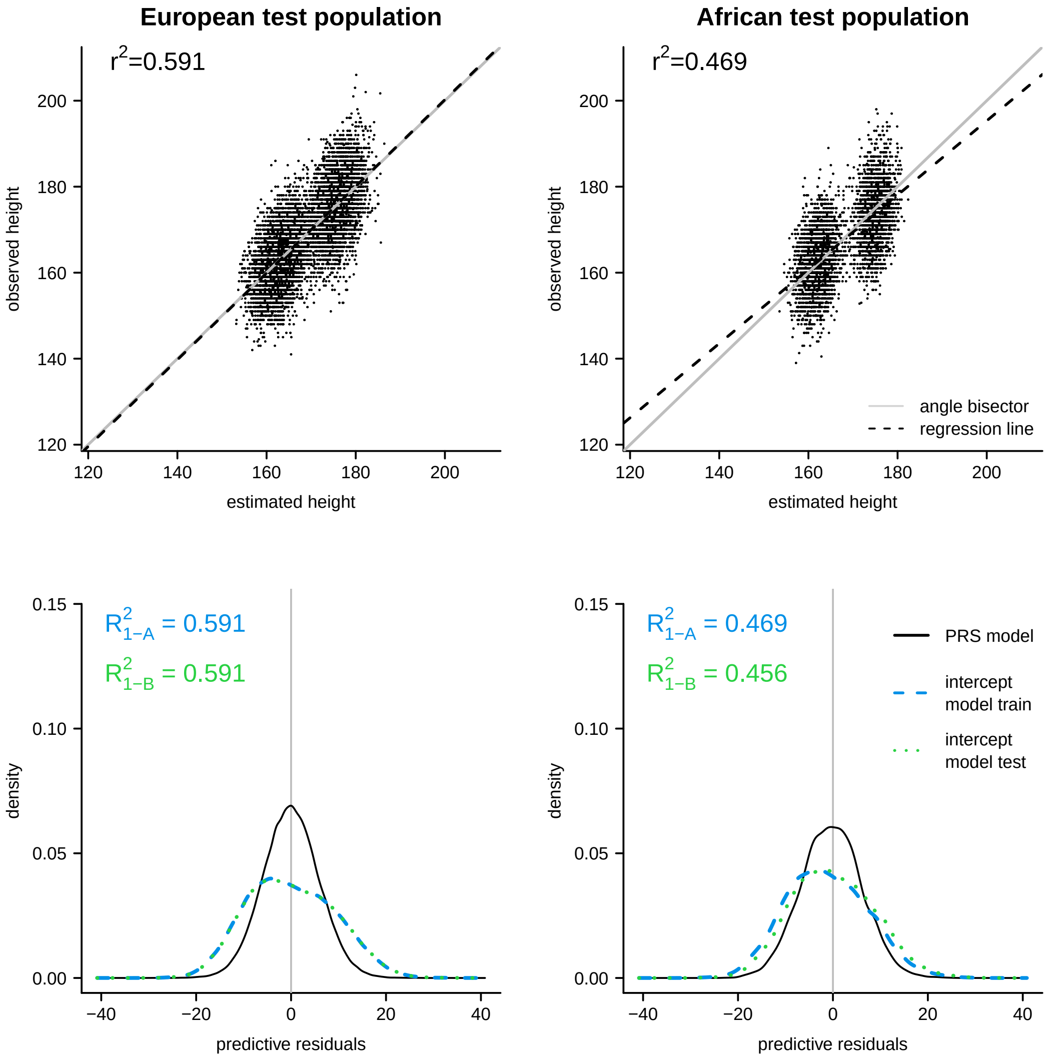

Supplementary Figure 3: Illustration of the three different R^2 definitions on test populations with European ancestry (left) and African ancestry (right) for the prediction of height, based on PRScs model derived on training data with European ancestry (including sex, age and first 10 genetic PCs). The scatterplots (top) show the association between predicted and observed heights, illustrating the R^2 definition based on the squared correlation (r^2). The kernel density plots (bottom) show the distributions of predictive residuals, illustrating the MSPE-based definitions of the R^2 with reference to intercept models on training data ($R^2_{1-\mathrm{A}}$) and test data ($R^2_{1-\mathrm{B}}$), respectively.

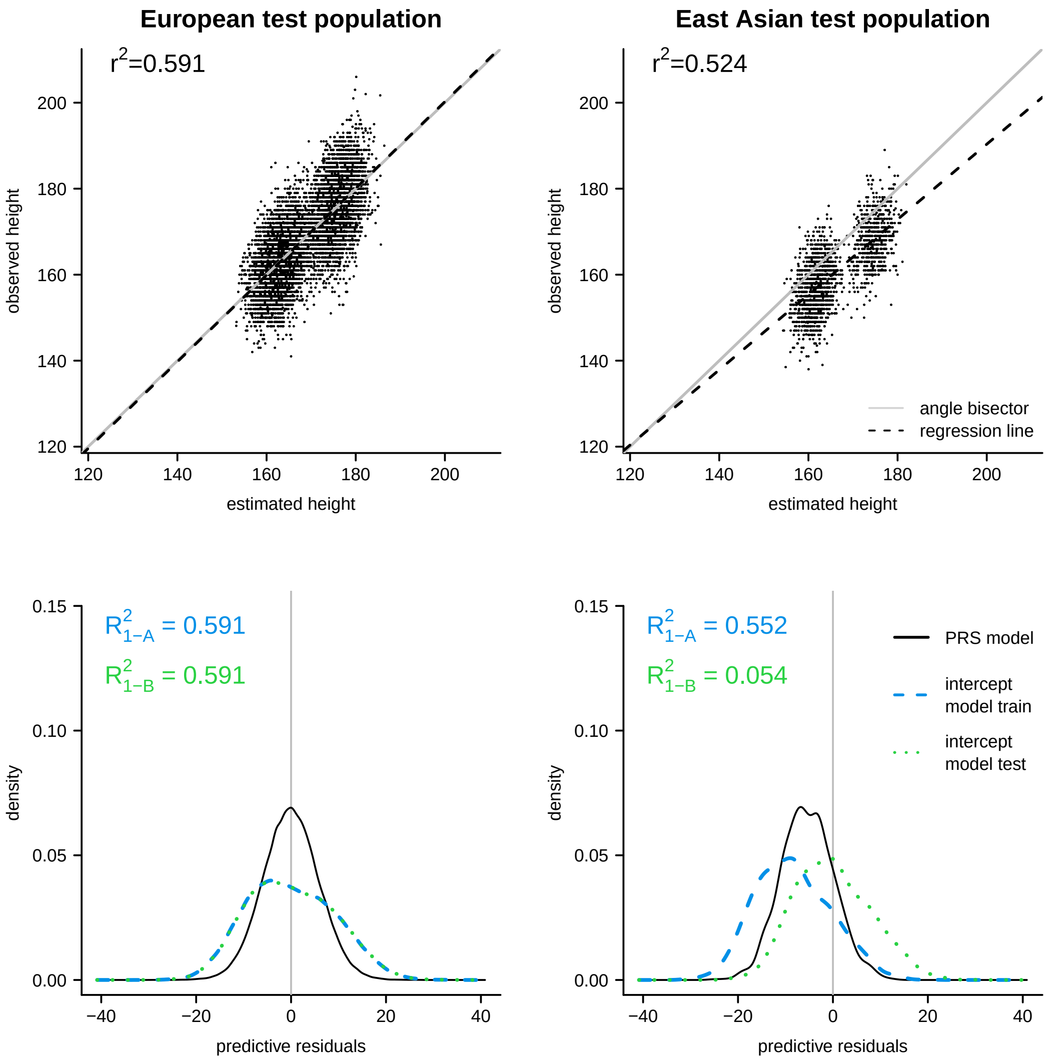

Supplementary Figure 4: Illustration of the three different R^2 definitions on test populations with European ancestry (left) and East Asian ancestry (right) for the prediction of height, based on PRScs model derived on training data with European ancestry (including sex, age and first 10 genetic PCs). The scatterplots (top) show the association between predicted and observed heights, illustrating the R^2 definition based on the squared correlation (r^2). The kernel density plots (bottom) show the distributions of predictive residuals, illustrating the MSPE-based definitions of the R^2 with reference to intercept models on training data ($R^2_{1-\mathrm{A}}$) and test data ($R^2_{1-\mathrm{B}}$), respectively.
